# Halofuginone inhibits TGF-β/BMP signaling and in combination with zoledronic acid enhances inhibition of breast cancer bone metastasis

**DOI:** 10.18632/oncotarget.21200

**Published:** 2017-09-23

**Authors:** Patricia Juárez, Pierrick G.J. Fournier, Khalid S. Mohammad, Ryan C. McKenna, Holly W. Davis, Xiang H. Peng, Maria Niewolna, Alain Mauviel, John M. Chirgwin, Theresa A. Guise

**Affiliations:** ^1^ Division of Endocrinology, Department of Medicine, Indiana University Purdue University at Indianapolis, Indiana, USA; ^2^ Ensenada Center for Scientific Research and Higher Education, Ensenada, Mexico; ^3^ University of Virginia, Charlottesville, Virginia, USA; ^4^ Institute Curie, Orsay, France; ^5^ INSERM U1021, Orsay, France; ^6^ CNRS UMR3347, Orsay, France; ^7^ Université Paris XI, Orsay, France

**Keywords:** halofuginone, zoledronic acid, bone metastases, TGF-β, BMP

## Abstract

More efficient therapies that target multiple molecular mechanisms are needed for the treatment of incurable bone metastases. Halofuginone is a plant alkaloid-derivative with antiangiogenic and antiproliferative effects. Here we demonstrate that halofuginone is an effective therapy for the treatment of bone metastases, through multiple actions that include inhibition of TGFβ and BMP-signaling.

Halofuginone blocked TGF-β-signaling in MDA-MB-231 and PC3 cells showed by inhibition of TGF-β–induced Smad-reporter, phosphorylation of Smad-proteins, and expression of TGF-β-regulated metastatic genes. Halofuginone increased inhibitory Smad7-mRNA and reduced TGF-β-receptor II protein. Proline supplementation but not Smad7-knockdown reversed halofuginone-inhibition of TGF-β-signaling. Halofuginone also decreased BMP-signaling. Treatment of MDA-MB-231 and PC3 cells with halofuginone reduced the BMP-Smad-reporter (BRE)_4_, Smad1/5/8-phosphorylation and mRNA of the BMP-regulated gene Id-1. Halofuginone decreased immunostaining of phospho-Smad2/3 and phospho-Smad1/5/8 in cancer cells *in vivo*.

Furthermore, halofuginone decreased tumor-take and growth of orthotopic-tumors. Mice with breast or prostate bone metastases treated with halofuginone had significantly less osteolysis than control mice. Combined treatment with halofuginone and zoledronic-acid significantly reduced osteolytic area more than either treatment alone. Thus, halofuginone reduces breast and prostate cancer bone metastases in mice and combined with treatment currently approved by the FDA is an effective treatment for this devastating complication of breast and prostate-cancer.

## INTRODUCTION

Despite the improvement in breast and prostate cancer treatment, 80 percent of the patients with advanced disease will develop bone metastases [1, 2]. The consequences of bone metastases are often devastating, and once tumors metastasize to bone, they are incurable and cause severe pain, fractures, spinal nerve compression and paralysis [3]. Cancer cells produce bone-resorbing factors leading to increased bone destruction. As a consequence, growth factors such as transforming growth factor (TGF-β) [4], bone morphogenetic proteins (BMPs) and others are released from the mineralized bone matrix. In turn, these factors increase tumor production of pro-angiogenic and pro-osteolytic factors establishing a feed-forward cycle, responsible for tumor growth and invasiveness in bone [5, 6]. TGF-β is a main regulator of this feed-forward cycle of bone metastasis [7, 8]. It stimulates the tumor production of bone-active factors (PTHrP, IL-11, ET-1) and prometastatic factors (VEGF, CXCR4, CTGF, MMPs), which disrupt normal bone remodeling and promote invasion, angiogenesis, and homing of tumor cells to bone [9].

Preclinical studies have shown that blockade of TGF-β signaling, using small molecule inhibitors of TGF-β receptor I kinase activity, reduced melanoma and breast cancer bone metastasis and prolonged mouse survival [10-12]. A neutralizing pan-TGF-β antibody reduced tumor burden and osteolytic lesions in a mouse model of bone metastasis [13], and overexpression of TGF-β-signaling inhibitor Smad7 in a mouse model delayed the establishment and the growth of melanoma bone metastases [14, 15].

Halofuginone is a natural product derivative that inhibits TGF-β signaling [16, 17], activates the amino acid starvation response [18] and has antiangiogenic and antiproliferative properties. Halofuginone has completed phase I and II clinical trials for the treatment of advanced solid tumors and HIV-related Kaposi’s sarcoma respectively [19, 20]. We and others have shown that halofuginone reduced metastasis and cancer progression in different models [21-24]. However its effects in breast and prostate cancer bone metastases have not been described. We hypothesized that halofuginone would be effective against breast and prostate cancer bone metastases and that combining halofuginone with the osteoclast inhibitor zoledronic acid, an FDA-approved treatment for bone metastases, would have additional efficacy against bone metastases.

Here, we tested the therapeutic potential of halofuginone to prevent breast and prostate cancer bone metastases. We explored its actions on TGF-β signaling and report for the first time that halofuginone inhibits BMP signaling. Finally we evaluated whether halofuginone combined with zoledronic acid was more effective than either drug alone to treat metastases to bone.

## RESULTS

### Halofuginone reduces breast and prostate cancer bone metastases in mice

**Figure 1:**
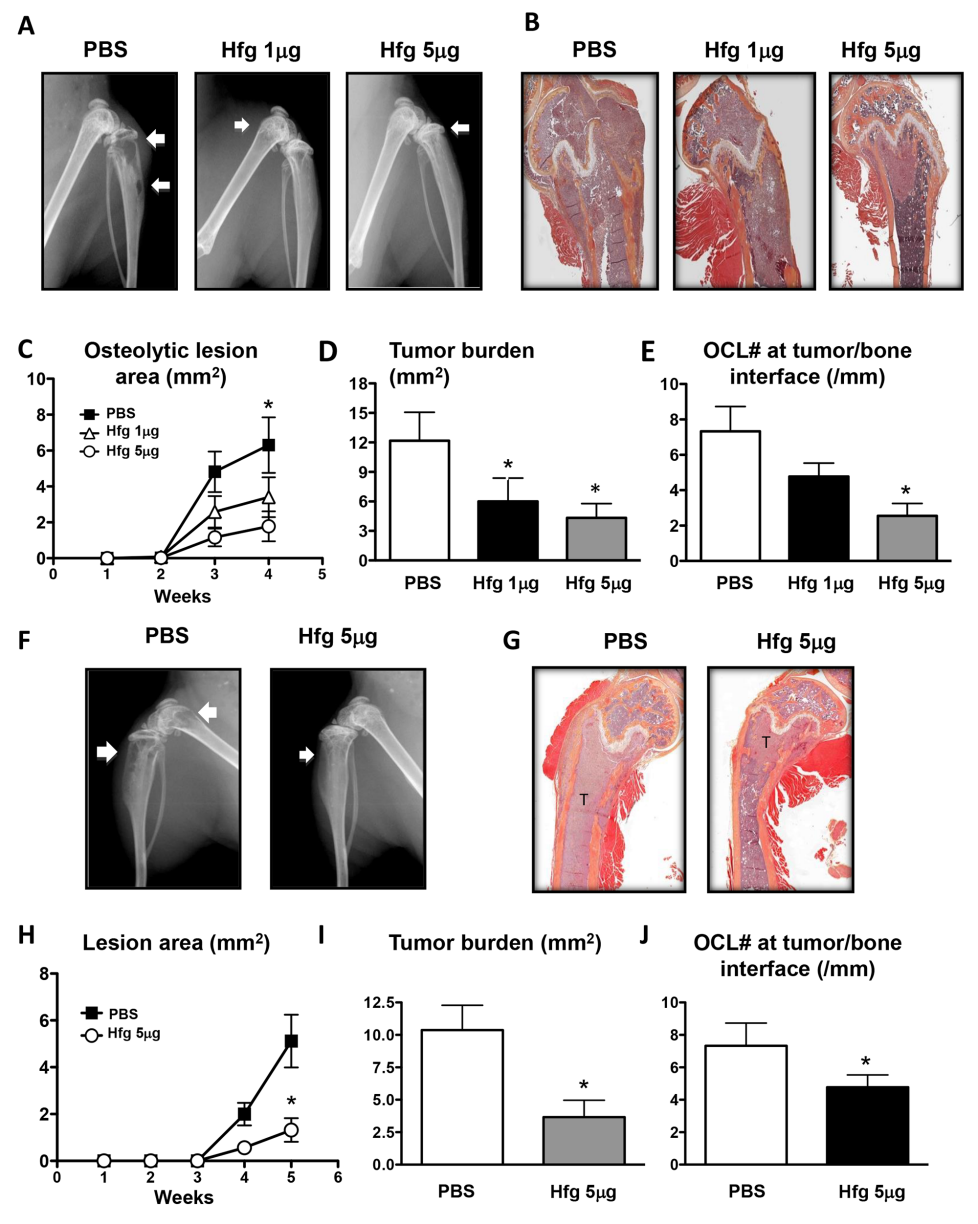
Halofuginone reduces breast and prostate cancer bone metastases (A, F) Representative radiography of the hindlimbs of female and male nude mice inoculated with MDA-MB-231 **(A-E)** or PC3 cells **(F-J)**, respectively, and treated with PBS or halofuginone (Hfg), 1 or 5µg/mouse/day for up to 5 weeks. Arrows indicate osteolytic lesions. (B, G) Representative histology of tibias with bone metastases. (C, H) Osteolytic area (mm^2^) measured on radiographs of hindlimbs of mice with bone metastases. * *P*<0.05 *vs* Hfg 1 or 5µg/mouse using one-way ANOVA with Bonferonni’s posttest. (D, I) Tumor burden area (mm^2^) measured by quantitative histomorphometry. (E, J) Osteoclast number at tumor-bone interface of hindlimbs of mice with bone metastases. *P<0.05 *vs* PBS using One-way ANOVA with Bonferroni’s post-test.

We evaluated the potential of halofuginone to treat breast and prostate cancer bone metastasis in a murine model of bone metastasis. High (5μg/mouse) and low doses (1μg/mouse) of halofuginone significantly reduced osteolytic lesion area compared to mice treated with PBS, when measured on radiographs (Figure 1A and 1C) and confirmed by histomorphometric analysis of the tumor area (Figure 1B and 1D). The number of osteoclasts at the tumor-bone interface was reduced in mice treated with halofuginone in a dose-dependent manner (Figure 1E).

Similar results were obtained using a prostate cancer bone metastasis model. Male nude mice were inoculated with PC3 prostate cancer cells and treated with halofuginone. As seen in the breast cancer bone metastases model, halofuginone (5μg/mouse) significantly reduced the osteolytic area and tumor burden compared to mice treated with vehicle (Figure 1F–1I). Osteoclast numbers at the tumor-bone interface were also significantly decreased (Figure 1J).

### Halofuginone inhibits TGF-β signaling

**Figure 2:**
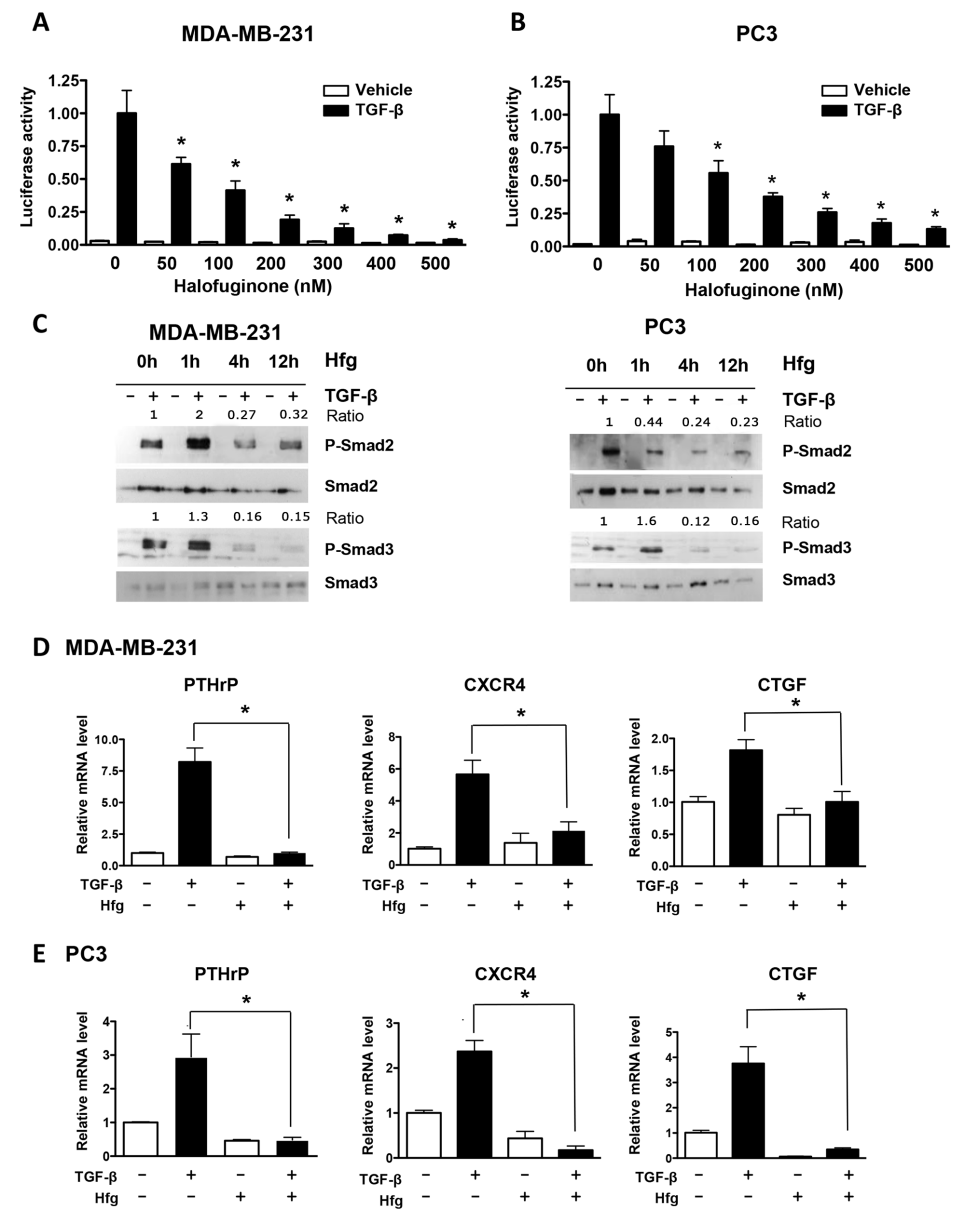
Halofuginone inhibits TGF-β signaling in MDA-MB-231 and PC3 cancer cells **(A, B)** Dual luciferase assay-(CAGA)_9_ reporter of MDA-MB-231 & PC3 cells treated with Hfg, followed by TGF-β (5ng/ml) for 24h. **(C)** Western blot analysis of breast and prostate cancer cells treated with 200nM Hfg for 1, 4 or 12h before adding TGF-β (5ng/mL, 30min) to assess Smad2 and Smad3 phosphorylation. **(D, E)** Expression of PTHrP, CXR4 and CTGF mRNAs in MDA-MB-231 and PC3 cells pretreated with Hfg (200nM) and treated or not with TGF-β (5ng/mL) and for 24h using quantitative real-time RT-PCR. *P<0.05 vs PBS using One-way ANOVA with Bonferroni’s post-test.

To understand the mechanism by which halofuginone inhibits bone metastases, we analyzed its effects on breast and prostate cancer cells transfected with pGL3-luc plasmid expressing firefly luciferase either constitutively or with a TGF-β-responsive reporter (CAGA)_9_-luc. Halofuginone decreased TGF-β–induced promoter activity in MDA-MB-231 and PC3 cancer cells in a dose-dependent manner (Figure 2A and 2B) while did not have any effect on the firefly luciferase constitutively active construct (Supplementary Figure 1). Next, we studied the TGF-β–induced phosphorylation of Smad2 and 3 proteins in cancer cells treated with halofuginone at different time points (1, 4 and 12h). Halofuginone treatment decreased Smad2 and Smad3 phosphorylation after 4 and 12h of treatment and as early as 1h for Smad3. Levels of total Smad2 and 3 were unchanged (Figure 2C). We then examined whether halofuginone could decrease the expression of TGF-β–induced genes that promote bone metastases [9]. TGF-β treatment alone increased mRNA levels of PTHrP, CXCR4 and CTGF in breast and prostate cancer cells. In contrast, cells treated with TGF-β in the presence of halofuginone had significantly lower levels of TGF-β-regulated mRNAs (Figure 2D and 2E).

### Halofuginone inhibits BMP signaling

**Figure 3:**
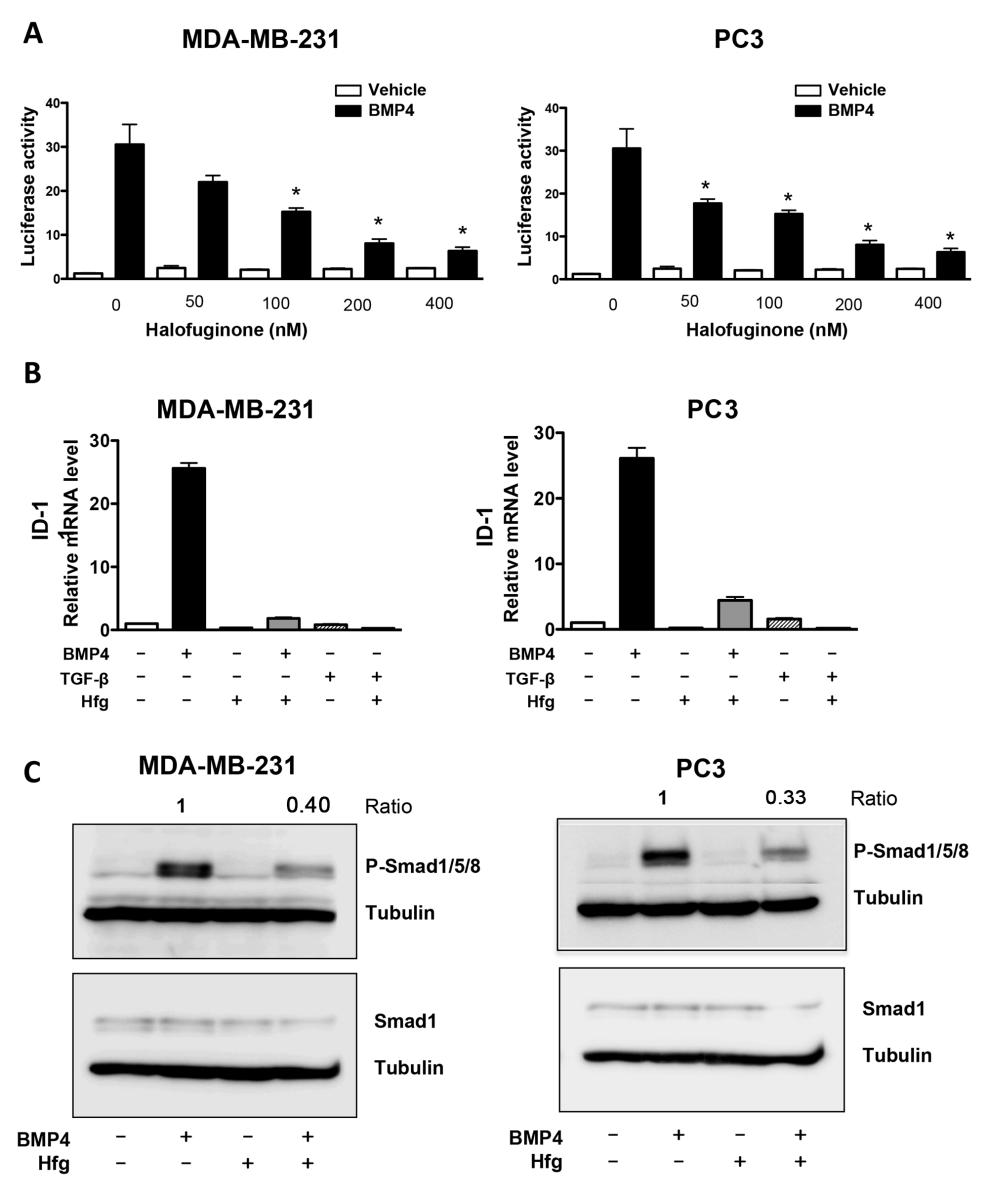
Halofuginone inhibits BMP signaling **(A)** Dual luciferase assay-(BRE)_4_ promoter of MDA-MB-231 & PC3 cells treated with Hfg followed by BMP4 (50ng/ml) for 24h. **(B)** Expression of ID1 mRNA in MDA-MB-231 and PC3 cells pretreated with Hfg (200nM) and treated or not with BMP4 (50ng/mL) and for 24h by qRT-PCR. **(C)** Western blot analysis for Smad1/5/8 phosphorylation of breast and prostate cancer cells pretreated with 200nM Hfg for 4h followed by BMP4 (50ng/mL) for 30min. *P<0.05 vs PBS using One-way ANOVA with Bonferroni’s post-test.

Bone morphogenetic proteins (BMPs) have been associated with cancer risk, and are also members of the TGF-β superfamily [27]. Therefore, we tested the effect of halofuginone on BMP signaling. We transfected breast and prostate cancer cells with a plasmid expressing firefly luciferase under the control of a BMP responsive reporter, (BRE)_4_. Cells were treated with BMP4 in the presence or absence of halofuginone. Halofuginone treatment significantly reduced the activity of BMP signaling responsive promoter in a dose-response manner (Figure 3A). In addition, we analyzed halofuginone effects on the mRNA expression of ID-1, a BMP-specific target gene. Only BMP4 increased ID-1 expression in MDA-MB-231 and PC3 cells, not a treatment with TGF-β (Figure 3B). Halofuginone reduced mRNA expression of ID-1 induced by BMP4 treatment (Figure 3B). Next we tested the effect of halofuginone on the activation of BMP-specific Smads. Halofuginone inhibited the phosphorylation of Smad1/5/8 in both MDA-MB-231 and PC3 cells, while total Smad1 was unchanged (Figure 3C). These results show for the first time that halofuginone inhibits BMP signaling.

### Halofuginone reduces intracellular mediators of TGF-β and BMP signaling *in vivo*

**Figure 4:**
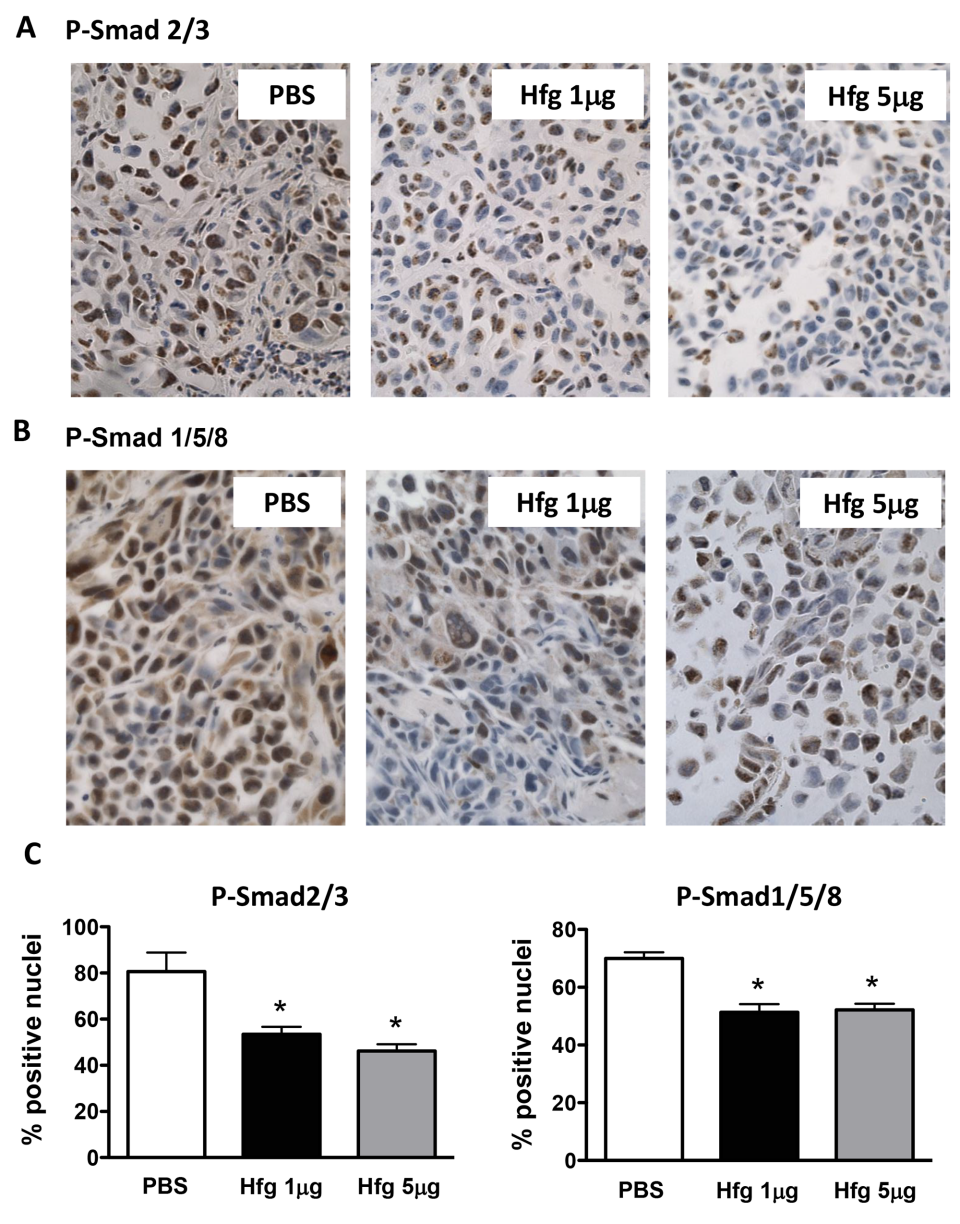
Halofuginone reduces TGF-β and BMP signaling in vivo Immunohistochemistry of **(A)** phospho-Smad2/3 and **(B)** phospho-Smad1/5/8 tumor tissue sections from limbs of mice with bone metastases caused by MDA-MB-231 cells and treated with Hfg (1μg/5μg) or PBS. **(C)** Quantitative analysis of nuclei positives for phosphorylated Smad2/3 or Smad1/5/8 in bone metastases. *P<0.05 vs PBS using One-way ANOVA Bonferroni’s post-test.

We asked whether halofuginone also reduced TGF-β and BMP signaling at sites of bone metastases*.* We used immunostaining of phosphorylated Smad2/3 to assess TGF-β signaling activity in bone metastases from MDA-MB-231 breast cancer cells in mice treated or not treated with halofuginone. Similarly to Kang *et al.*, we found that phospho-Smad2/3 mainly localized in the nuclei of cancer cells closer to the tumor-bone interface indicating a gradient of TGF-β released from the bone matrix [4]. There was a significant reduction of nuclear phospho-Smad2/3 in cancer cells when mice were treated with halofuginone, showing a decrease of TGF-β signaling activity (Figure 4A and 4C). Similarly, BMP signaling activity *in vivo* was assessed by immunostaining using an antibody against phosphorylated Smad1/5/8. In mice treated with halofuginone (1 and 5µg/day) there was an inhibition of BMP signaling activity in breast cancer cells in bone as measured by phospho-Smad1/5/8-positive nuclei (Figure 4B and 4C).

### Knockdown of Smad7 does not prevent inhibition of TGF-β signaling by halofuginone

**Figure 5:**
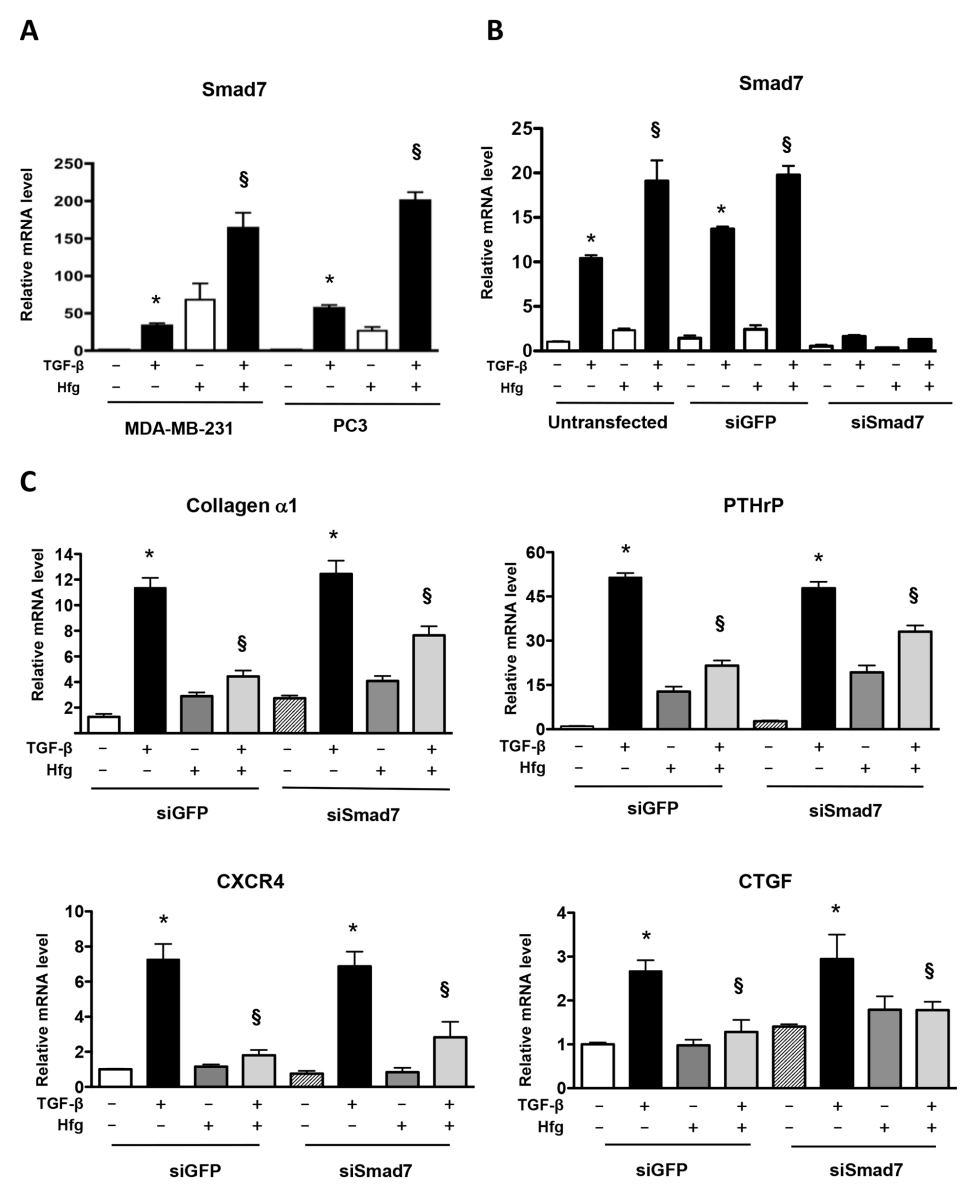
Knockdown of Smad7 does not prevent inhibition of TGF-β by halofuginone **(A)** Smad7 mRNA levels analyzed by qRT-PCR in MDA-MB-231 and PC3 cells treated with 200nM Hfg for 4h followed by 5ng/ml TGF-β for 60 min. **(B)** MDA-MB-231 cells transfected or not transfected with siRNA against Smad7 or GFP as a control. Smad7 mRNA was quantified by qRT-PCR. **(C)** Quantification of mRNA levels of Collagen α1 and pro-metastatic genes PTHrP, CXCR4 and CTGF in MDA-MB-231 cells with or without a knockdown of Smad7 or TGF-β (5ng/mL) and Hfg (200nM) for 24h. *P<0.05 vs PBS, § P<0.05 vs Hfg using One-way ANOVA with Bonferroni’s post-test.

Halofuginone induces Smad7 mRNA expression, a negative regulator of TGF-β signaling [17]. Inhibition of TGF-β signaling by halofuginone could be mediated by Smad7. We tested whether knockdown of Smad7 could overcome TGF-β signaling inhibition caused by halofuginone. TGF-β increased Smad7 mRNA in MDA-MB-231 and PC3 cells. Halofuginone treatment alone induced Smad7 mRNA mainly on MDA-MB-231 cells, however combined with TGF-β enhanced the increase of Smad7 in both breast and prostate cell lines (Figure 5A). We used a combination of 2 siRNAs to decrease Smad7 mRNA by 80% in the presence of TGF-β and halofuginone, when compared to untransfected cells or to cells transfected with a GFP siRNA control (Figure 5B). Real-time PCR of MDA-MB-231 samples showed that Smad7 knockdown did not prevent the decrease of TGF-β regulated genes (collagen α1, PTHrP, CXCR4, CTGF) caused by halofuginone (Figure 5C). Therefore Smad7 does not mediate the inhibition of TGF-β signaling by halofuginone in MDA-MB-231.

### Halofuginone inhibits TBRII translation

**Figure 6:**
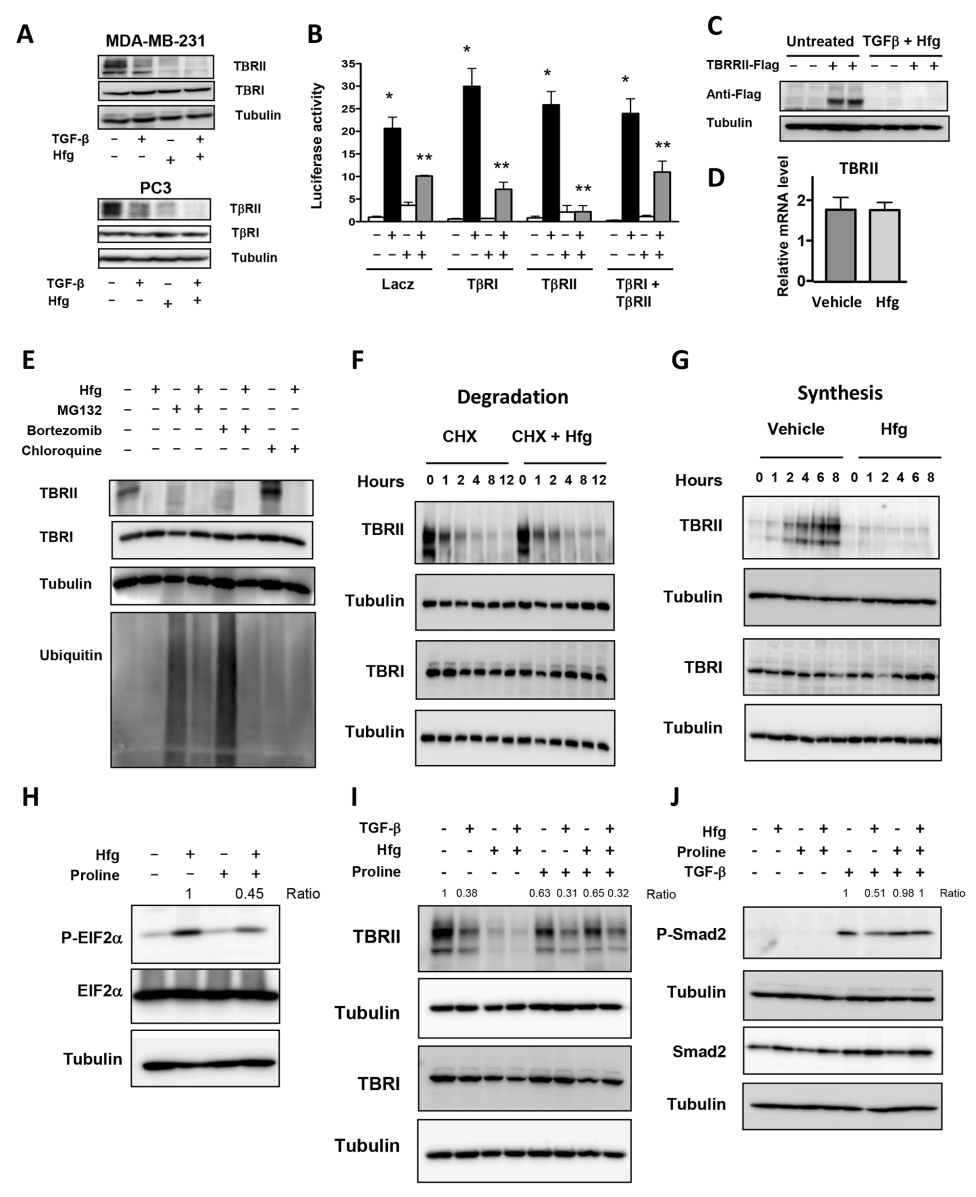
Halofuginone inhibits TBRII synthesis by activating the amino acid starvation response **(A)** Western blot analysis of MDA-MB-231 & PC3 cells for TGF-β type I and II receptors (TβRI and TβRII) after a 24h-treatment with 5ng/ml TGF-β and 200nM Hfg. **(B)** Dual luciferase assay-(CAGA)_9_ reporter of MDA-MB-231 & PC3 cells transfected or not with TβRI, TβRII or both, 24h later cells treated with 200nM Hfg and 5ng/ml TGFβ for 24h. **(C)** Western blot of MDA-MB-231 cells transfected with pRK5-TβRII-Flag plasmid treated with vehicle or 200nM Hfg and 5ng/ml TGF-β. **(D)** TBRII mRNA levels analyzed by qRT-PCR in MDA-MB-231 cells treated with 200nM Hfg for 4h followed by 5ng/ml TGF-β for 30 min for 24 hrs. **(E)** MDA-MB-231 cells treated with 5μM MG132, 1μM bortezomib or 10μM chloroquine for 24h lysed and tested for TBRII, TBRI, Tubulin and Ubiquitin. **(F-G)** Time course of TGF-β receptor analysis, MDA-MB-231 cells treated with 1μM cycloheximide en the presence of 200nM Hfg (F) or pretreated with cycloheximide overnight followed by 200nM Hfg (G). **(H)** Proline (1μM) was added to the media of MDA-MB-231 cells treated with Hfg or 5ng/ml TGF-β alone or with 100nM Hfg. **(I, J)** Western blot of MDA-MB-231 cells treated with 100nM Hfg alone or with 1μM proline for 4h, to detect Phospho-EIF2 and EIF2. J) After 30 min of TGF-β treatment, Phospho-Smad2 and Smad2 proteins are showed. All western blot experiments were normalized with tubulin. * P<0.05 vs PBS, **P<0.05 vs TGF-β. One-way ANOVA Bonferroni’s post-test.

A second mechanism proposed for TGF-β signaling inhibition by halofuginone is through down-regulation of TGF-β receptor II (TBRII) [17]. Figure 6A shows that halofuginone treatment of breast and prostate cancer cells reduces TBRII protein levels, while TGF-β receptor I (TBRI) protein levels were unaffected. Transfection of MDA-MB-231 with plasmids expressing TBRII and TBRI alone or in combination did not reverse halofuginone-induced inhibition of TGF-β signaling (Figure 6B). Besides, Western blot analysis showed that exogenous Flag-TBRII protein was not detected in MDA-MB-231 treated with halofuginone (Figure 6C). RT-PCR indicated that halofuginone did not have any effect on TBRII mRNA suggesting that halofuginone effect on TBRII is post-transcriptional.

We investigated first whether halofuginone increases TBRII degradation. Neither the proteasome inhibitors MG132 and bortezomib, nor chloroquine, which inhibits lysosomal degradation, prevented the halofuginone-induced decrease of TBRII (Figure 6E). Next, we treated our cells with cycloheximide to stop de novo protein synthesis and assayed TGF-β receptor degradation by Western blot. After addition of cycloheximide, TBRII was quickly degraded and addition of halofuginone did not increase the degradation rate of TBRII (Figure 6F). TBRI appeared more stable, and its levels remained unchanged after 12h of treatment (Figure 6F). To test whether halofuginone affects TBRII synthesis, we treated cells with cycloheximide overnight to stop de novo protein synthesis and eliminate endogenous TBRII. Cycloheximide was then removed and new TBRII protein was detected 2h later. Addition of halofuginone completely prevented the synthesis of TBRII protein (Figure 6G). These results show that halofuginone inhibits TBRII protein translation.

### Proline reverses inhibitory effects of halofuginone on TGF-β signaling

Keller *et al*, have shown that halofuginone is a potent competitive inhibitor of the glutamyl-prolyl-tRNA-synthetase that adds proline to its tRNA [28]. Accumulation of amino acid-free tRNA triggers the amino acid response (AAR) and the phosphorylation of the translation initiation factor EIF2α. Halofuginone induced the phosphorylation of EIF2α, which was prevented by the addition of proline (Figure 6H).

We therefore examined whether proline supplementation would reverse the inhibitory effects of halofuginone on TBRII. Proline reversed TBRII suppression in cells treated with halofuginone for 24h in the presence or absence of TGF-β (Figure 6I). Inhibition of Smad2 phosphorylation by halofuginone was also rescued by the addition of proline (Figure 6J) indicating that halofuginone inhibits TGF-β signaling is mediated by the AAR.

### Halofuginone reduces orthotopic tumor growth and in combination with zoledronic acid enhances inhibition of bone metastasis

**Figure 7:**
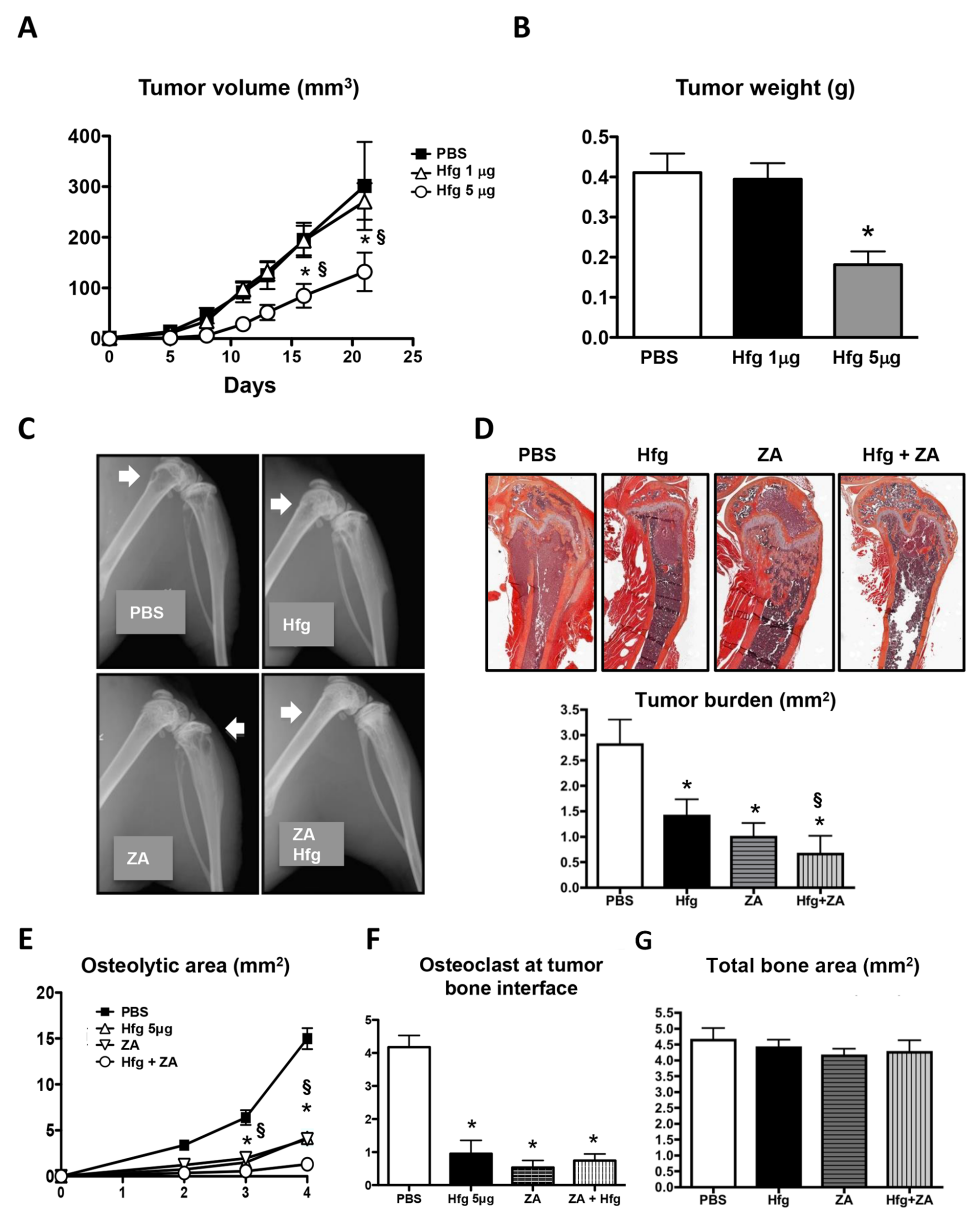
Halofuginone reduces orthotopic tumor growth and in combination with zoledronic acid enhances inhibition of bone metastases **(A)** Volume and **(B)** weight of MDA-MB-231 tumors in the mammary fat pad of female nude mice treated with halofuginone (Hfg, 1 or 5μg/mouse) or PBS. **(C)** Representative radiography of hind limbs of female nude mice inoculated with MDA-MB-231 cells and treated with PBS, Hfg (5μg/mouse/day), zoledronic acid (ZA, 5μg/kg/3x per wk) or Hfg and ZA combined. **(D)** Representative images of tumor burden and histomorphometric quantification. **(E)** Quantification of osteolytic area on radiography. **(F)** Quantification of the osteoclast number at the tumor bone interface and **(G)** Total bone area *P<0.05 vs PBS, § P<0.05 vs Hfg using One-way ANOVA with Bonferroni’s post-test.

To investigate the effects of halofuginone on primary tumors, we inoculated breast cancer cells in the mammary fat pad (MFP) of female nude mice and treated them with two different doses of halofuginone (1 or 5μg/mouse/day). Mice treated with the high dose of halofuginone showed a significant reduction in the tumor volume and tumor weight compared to the control mice. Low dose halofuginone treatment did not affect the growth of MFP tumors (Figure 7A and 7B).

We next evaluated combined treatment with halofuginone and zoledronic acid, an inhibitor of bone resorption approved by the FDA for the treatment of bone osteolysis. We inoculated female nude mice with breast cancer cells and treated them during 4 weeks with PBS as a placebo or with halofuginone (5μg/mouse/day) or zoledronic acid (5μg/kg/3X per week) alone or combined. Halofuginone and zoledronic acid treatments alone significantly reduced to a similar extent osteolytic area on radiographs (Figure 7C and 7E). Combined treatment further decreased the area of osteolysis when compared to mice treated with either agent alone. Histomorphometric analysis of the tumor burden confirmed that halofuginone and zoledronic acid alone decreased the tumor burden area when compared to PBS-treated-mice and that the combined treatment was more efficient at decreasing tumor area than halofuginone alone (Figure 7D). Analysis of TRAP+ osteoclasts showed that halofuginone and zoledronic acid alone or combined significantly reduced osteoclast number (Figure 7F). Total bone area was not affected by any of the treatments (Figure 7F).

## DISCUSSION

Bone metastasis is a very frequent complication of patients with advanced breast and prostate cancer. Preclinical models have demonstrated that blockade of TGF-β signaling is effective to prevent bone metastasis. In this study, we found that halofuginone reduced breast and prostate cancer metastases *in vivo* and this reduction was enhanced by combination with zoledronic acid in a mouse model of breast cancer bone metastasis. We previously demonstrated the potential of halofuginone to reduce bone metastasis in a mouse model of melanoma [24]. In a similar manner, halofuginone significantly reduced tumor burden and osteolytic lesion area in both models of breast and prostate cancer bone metastasis. This inhibition was accompanied by a reduction of the number of osteoclasts at the tumor-bone interface. Halofuginone inhibited cell proliferation of breast and prostate cancer cells *in vitro* (data not shown) and reduced tumor growth in mice at the orthotopic site, showing that its effects are not limited to bone; unlike the effects of other TGF-β inhibitors, including SD208 and overexpression of Smad7 in melanoma cells [12, 15]. The effects of halofuginone against bone metastases are consistent with an inhibition of TGF-β signaling *in vivo*, as shown by the decrease of phosphorylated Smad2/3 in the nuclei of cancer cells, and a subsequent decrease of TGF-β-regulated prometastatic genes in tumor cells. However we can not exclude that halofuginone has direct effect on bone cells such as osteoclasts and osteoblasts.

*In vitro* studies confirmed that halofuginone inhibited TGF-β signaling in breast and prostate cancer cells as shown by: a) reduction of TGF-β receptor II protein, b) inhibition of Smad intracellular mediators, c) inhibition of a TGF-β-responsive reporter and d) reduction of prometastatic TGF-β-regulated genes.

We explored the effects of halofuginone on bone morphogenetic protein (BMP) signaling, since BMPs are members of the TGF-β superfamily. BMPs have been linked to cancer predisposition and are aberrantly expressed in multiple malignancies [27, 29]. The role of BMPs in cancer is still emerging and vary from one BMP ligand and tumor type to another, resembling the dual role of TGF-β as a suppressor of tumorigenesis and promoter of metastasis [30, 31]. *In vitro*, breast and prostate cancer cells treated with halofuginone showed inhibition of BMP signaling demonstrated by inhibition of BMP-responsive promoter, inhibition of Phospho-Smad1/5/8 and reduction of ID1, a regulator of cancer cell growth and migration [32]. Consistent with the *in vitro* data, tumors of mice with breast cancer bone metastasis had a reduction in phospho SMAD1/5/8-positive nuclei, suggesting an inhibition of BMP signaling in the bone metastatic microenvironment of mice treated with halofuginone. Whether halofuginone effects on TGFβ and BMP signaling are correlated or independent of each other needs to be determined.

Although halofuginone inhibition of TGF-β signaling has been described before [17, 33], its precise mechanisms of action remain unknown. It was proposed that the inhibition of TGF-β signaling by halofuginone is mediated by induction of Smad7, a negative regulator of TGF-β signaling, and down regulation of TGF-β receptor II (TβRII) [17]. In breast and prostate cancer cells, halofuginone increased Smad7 mRNA; however knockdown of Smad7 did not prevent halofuginone inhibition of TGF-β-regulated genes. When studying the regulation of TBRII, TGFβ itself caused a significant reduction in the amount of TBRII protein, while co-treatment with halofuginone appears to enhance that effect. Halofuginone also prevented the expression of endogenous as well as exogenous TBRII protein without affecting mRNA levels, suggesting that halofuginone effects are post-transcriptional. Proteasome and lysosome inhibitors, and a cycloheximide-chase assay suggested that halofuginone does not regulate TBRII degradation. Analysis of newly synthesized TBRII protein in cells confirmed that halofuginone prevents TBRII expression at the translational level. Sundrud *et al* recently showed that halofuginone activates the amino acid starvation response pathway and inhibits differentiation of TH17 cells [18]. Amino acid restriction results in accumulation of amino acid-free tRNAs and the subsequent inhibition of the translation machinery. Halofuginone triggers the amino acid starvation pathway by inhibiting prolyl tRNA synthase activity, which can be reversed by the addition of exogenous proline [28]. Proline addition effectively prevented the halofuginone-induced activation of the AAR pathway as shown by the decreased phosphorylation of EIF2. Proline also rescued the inhibition of TBRII synthesis caused by halofuginone and restored TGF-β signaling pathway. These results indicate for the first time that halofuginone inhibits TGF-β signaling by activating the amino acid starvation pathway that stops protein translation and results in the quick depletion of TBRII in cancer cells. Whether halofuginone activates the amino acid starvation pathway in cancer cells at the site of bone metastases in our mouse model remains to be determined.

Lastly, we evaluated the anti-tumor efficacy of halofuginone in combination with the bisphosphonate, zoledronic acid, in a mouse model of breast cancer bone metastasis. Bisphosphonates are a palliative therapy available for cancer patients with skeletal-related symptoms. Nitrogen-containing bisphosphonates block osteoclastic bone resorption by inhibiting the mevalonate pathway, leading to osteoclast apoptosis [34]. They may also have direct anti-tumor effects, including inhibition of tumor growth, tumor invasion and anti-angiogenic activity [35, 36]. Bisphosphonates reduce the availability of TGF-β to tumor cells through inhibition of bone matrix destruction [4, 37]. In our study, combined treatment with halofuginone and zoledronic acid decreased the tumor burden and osteolytic lesion area in mice more effectively than either treatment alone. The combined treatment was associated with a reduction of the osteoclast number at the tumor-bone surface. The beneficial effects of the combined therapy may be due to the inhibition of TGF-β signaling and direct effects on tumor of both molecules, but the precise mechanism remains to be established. Interestingly, we also observed slightly less trabecular bone in mice treated with the combined treatment compared to mice treated with zoledronic acid alone. Considering that zoledronic acid efficiently inhibited bone resorption in our model, the lower amount of trabecular bone suggests that halofuginone also has an effect on bone formation. This would be consistent with the inhibition of BMP signaling induced by halofuginone since BMPs are a main regulator of bone formation. Experiments to determine the effect of halofuginone on bone formation in normal bone are ongoing.

In summary, halofuginone reduces breast and prostate cancer bone metastasis and inhibits multiple targets including TGF-β and BMP signaling pathways as shown by inhibition of TGF-β and BMP regulated prometastatic genes. It also has direct effects on the growth of cancer cells. The combination of halofuginone and zoledronic acid was more effective to decrease osteolytic bone lesions, suggesting that patients with bone metastasis may be treated with lower dose of zoledronic acid when in combination with halofuginone reducing the risk of side effects associated with bisphosphonates.

## MATERIALS AND METHODS

### Cell cultures and reagents

MDA-MB-231 breast and PC3 prostate cancer cells (both from the American Type Culture Collection) were cultured in DMEM or RPMI media, respectively, supplemented with 10% FBS and antibiotics. All cells were grown at 37°C with 5% CO_2_ in a humidified chamber. Halofuginone (dl-trans-7-bromo-6-chloro-3-[3-(3-hydroxy-2piperidyl) acetonyl]-4(3H)-quinazolinone hydrobromide) was a gift from Intervet Innovation GmbH. For *in vitro* studies, halofuginone stock solutions (2mg/ml) were prepared in lactic acid buffer (0.44M, pH 4.3) and stored at -20°C [24]. For *in vivo* experiments, halofuginone (1 or 5μg/0.1 ml) was resuspended in PBS. Zoledronic acid (Novartis) stock solution was prepared in 0.15M NaCl. Recombinant human TGF-β1 and BMP-4 were purchased from R&D Systems Inc. pRK5-TGFRII-Flag plasmid (31719) was obtained from Addgene.

### Western blot analysis

Cells were pretreated with halofuginone (200nM) for 4h followed by TGF-β (5ng/ml) or BMP4 (200ng/ml) for 30min for phospho-Smad protein analysis, and 24h for TGF-β receptor analysis. Proteins were separated by SDS-PAGE and transferred onto a Hybond™-P membrane (GE Healthcare Life Sciences). Membranes were blocked in TBST-milk (5%) for 1h, incubated overnight with primary antibody and for 1h with peroxidase-conjugated secondary antibody. Protein detection used Western Chemiluminescent HRP substrate (EMD Millipore). Antibodies against Smad2/3, Smad1, phospho-Smad2/3, phospho-Smad1/5/8, EIF2α and phospho-EIF2α were purchased from Cell Signaling. Anti-α-tubulin (Sigma) was used for normalization.

### Gene expression analysis

Cells were pretreated with halofuginone (200nM) for 4h followed by TGF-β (5ng/ml) or BMP4 (200ng/ml) for 24h in basal media-FBS (0.2%). Cells were lysed in Trizol (Invitrogen). Total RNA was isolated using RNeasy kit (Qiagen) and reverse transcribed using Superscript II (Invitrogen). qRT-PCR was performed using QuantiTect SYBR Green PCR Kit (Qiagen) and processed in a MyiQ™ Single-Color Real Time PCR detection system (BioRad) for 40 cycles (95°C for 15 sec/58°C for 30 sec/72°C for 30sec) after an initial 15min incubation at 95°C. Primers were optimized for qRT-PCR amplification efficiency 100±5% (for sequences see Supplemental Material). Data of triplicates samples were analyzed by the ΔΔCt method [25].

### Dual-luciferase assay

For TGF-β-responsive promoter studies, cells were transfected with pGL3-luc constructs (Promega) expressing firefly luciferase either constitutively or under the control of a Smad3/Smad4-specific promoter (CAGA)_9_ [26]. To study TGF-β receptors, cells were cotransfected with (CAGA)_9_ promoter, pcDNA3-TBRI and pcDNA3-TBRII (kind gift from Dr. J. Massague, Memorial Sloan-Kettering Cancer Center) alone or combined. For BMP studies, cells were transfected with a pGL3 plasmid containing the (BRE)_4_ luciferase reporter based on the mouse Id1 promoter and responsive to BMP (generously provided by Dr Peter Ten Dijke). phRL-CMV plasmid constitutively expressing *Renilla* luciferase (Promega) was used for normalization in all analyses. Twenty-four hours later, cells were treated with different concentrations of halofuginone (50-500nM) for 4h in basal medium supplemented with 0.2% FBS, followed by TGF-β (5ng/ml) or BMP4 (50ng/ml) for 24h. Cells were lysed using Passive Lysis Buffer (Promega), and analyzed for luciferase activity using the Dual-Luciferase Reporter Assay System (Promega) and a Synergy MX plate reader (Biotek).

### Knockdown of Smad7

MDA-MB-231 cells were transfected with a mix of siRNAs designed against Smad7 (GeneSolution) to maximize knockdown efficiency (for sequences see Supplemental Material). Cells at 80% of confluence were transfected with 20nM of each siRNAs-Smad7. Twenty-four hours later, cells were pretreated with halofuginone (200nM) for 4h in basal media supplemented with 0.2% FBS, followed by TGF-β (5ng/ml) for 24h. Cells were lysed and total RNA analyzed by qRT-PCR for Smad7 and TGF-β regulated genes.

### Protein degradation and synthesis assay

MDA-MB-231 cells were treated with proteasome inhibitors, MG132 (5μM) and Bortezomib (1μM), or lysosome inhibitor, chloroquine (10μM), for 24h, in the presence or absence of 200nM halofuginone. For protein degradation, cells were treated with cycloheximide (1μM) alone or with halofuginone (100nM) for 1-12h. To evaluate protein synthesis, cells were treated with cycloheximide (1μM) overnight, next day cells were washed twice with PBS followed by vehicle or halofuginone (100nM) treatment for 1-8h. Cells were lysed and Western blot analysis for TGF-β receptors was performed.

### Proline rescue experiments

MDA-MB-231 cells were pretreated with halofuginone (100nM) alone or with 1μM proline (Sigma Aldrich) for 4h in DMEM media. Followed by TGF-β (5ng/ml) treatment in DMEM (0.2% FBS), for 30min for phospho-Smad protein analysis or 24h for TGF-β receptors. For phospho-EIF2α and EIF2α protein analysis, cells were treated with halofuginone (100nM) alone or with proline for 4h. Cells lysates were assayed by Western blot.

### Animal studies

Animal experiments were performed at the University of Virginia in Charlottesville, Virginia and Indiana University. Animal protocols were in accordance with the national and international guidelines and approved by the respective Institutional Animal Care and Use Committees.

### Mammary fat pad tumor model

MDA-MB-231 cells (10^6^ cells per 100μl of PBS) were inoculated into the upper mammary fat pad of 5 weeks-old female nude mice. When palpable tumors were detected, the mice were divided into 3 groups (n=10) to receive either halofuginone (1 or 5μg/mouse/day) or PBS treatment daily by i.p. injection. Tumor volume was measured with a caliper three times per week and calculated from the formula: tumor volume = 4/3 π x L/2 (W/2)^2^, where L and W represent mid-axis length and width, respectively.

### Bone metastasis mouse model

Intracardiac inoculation of tumor cells was performed as previously described [14]. Briefly, MDA-MB-231 or PC3 cancer cells were trypsinized and resuspended in PBS to a final concentration of 10^6^ cells per milliliter. Five-weeks old female or male mice (n=12) were anesthetized and inoculated into the left ventricle with MDA-MB-231 or PC3 cells 10^5^ in 100µl/mouse. Halofuginone (1 or 5μg/mouse), or PBS were administered daily by i.p. injection two days prior to tumor cell inoculation and continued throughout the experiment. The development of osteolytic lesions was followed with a Faxitron MX-20 X-ray machine (Faxitron X-ray Corporation). Lesion area was confirmed with histology and quantified using MetaMorph analysis system software (Universal Imaging Corporation). Combined treatment with halofuginone and zoledronic acid followed similar experimental approach, halofuginone (5μg/mouse) and PBS were administrated daily by i.p. injection while zoledronic acid (5μg/kg) was administered three times per week s.c. throughout the experiment.

### Bone histology & histomorphometry

Bones from the forelimbs and hindlimb were collected upon euthanasia, fixed in 10% formalin for 48h, decalcified in 10% EDTA for 2 weeks, and embedded in paraffin. Tissue sections from tibia and femur were cut using an automated Microm HM 355S microtome (Thermo Fisher Scientific) and stained with hematoxylin and eosin (H&E) or for TRAP (tartrate-resistant acid phosphatase) activity. Bright field images were captured using a Q-Imaging Micropublisher Cooled CCD color digital camera (Vashaw Scientific Inc., Washington, DC, USA) on a Leica DM LB compound microscope (Leica Microsystem, Bannockburn, IL, USA). Histomorphometric analysis of tumor burden area, defined as area of bone occupied by cancer cells, and total bone area was performed using BIOQUANT OSTEO software (Image Analysis Corporation). Osteoclast numbers at the tumor bone interface were quantified on TRAP-stained sections of femur and tibia at 400X magnification.

### Immunohistochemistry

Immunohistochemical analysis was performed on decalcified paraffin-embedded tissue sections. Tissue sections were deparaffinized and treated with 3% hydrogen peroxide and trypsin for antigen retrieval (Invitrogen). Blocking of non-specific binding sites was done using normal goat serum (10%) and an avidin/biotin blocking kit (Vector Laboratories) before probing using antibodies against phospho-Smad2/3 or phospho-Smad1/5/8 (Cell Signaling) at 1:500 dilution. After incubation with a biotin-conjugated anti-rabbit antibody (EMD Millipore) and a streptavidin-peroxidase conjugate (Vector Laboratories), slides were stained using 3,3-diaminobenzidine substrate kit (Vector Laboratories) and counterstained with hematoxylin. The percentage of phospho-Smad positive nuclei per field was quantified. Five fields (200X) per animal were analyzed.

### Statistical analysis

Results are expressed as mean ± SEM. Samples were analyzed in triplicate. Differences between groups were analyzed by one-way ANOVA or two-way ANOVA followed by Bonferroni’s post-test. All the data were analyzed using Graphpad Prism v4.0 software (GraphPad Sofware, Inc). P<0.05 was considered significant.

### Supplementary information

A single pdf includes Supplementary Methods for the design of real-time PCR primers and siRNAS Smad7 sequences, and one Supplementary Figure showing the halofuginone effect in a CMV constitutive activated promoter.

## SUPPLEMENTARY MATERIALS FIGURE



## Abbreviations

Hfg: halofuginone
TGF-β: Transforming growth factor beta
BMP: bone morphogenetic protein
TBRI: TGF-β type I receptor
TBRII: TGF-β type II receptor
qRT-PCR: quantitative real time PCR
PTHrP: parathyroid hormone-related protein
CXCR4: chemokine receptor type 4
CTGF: connective tissue growth factor
OCL: osteoclast
ZA: zoledronic acid
