# Supplementary material for: Halofuginone inhibits TGF-β/BMP signaling and in combination with zoledronic acid enhances inhibition of breast cancer bone metastasis
Source: Oncotarget. 2017 Sep 23;8(49):86447–62. doi: 10.18632/oncotarget.21200 (PMC5689697; doi:10.18632/oncotarget.21200)
Supplement: Supplementary file 1 [file oncotarget-08-86447-s001.pdf]

Halofuginone inhibits TGF-β/BMP signaling and in combination with zoledronic acid enhances inhibition of breast cancer bone metastasis

SUPPLEMENTARY MATERIALS

Primer sequences for experiments

Primer	Sense (5′—3′)	Antisense (5′—3′)
PTHrP	ACTCGCTCTGCCTGGTTAGA	GGAGGTGTCAGACAGGTGGT
CXCR4	CCGTGGCAAACTGGTACTTT	GACGCCAACATAGACCACCT
CTGF	GCTACCACATTTCCTACCTAGAAATCA	GACAGTCCGTCAAAACAGATTGTT
Collagen α1	CCTGGATGCCATCAAAGTCT	CGCCATACTCGAACTGGAAT
ID1	CTCTACGACATGA	TGCTCACCTTGCGGTTCTG
Smad7	CCAACTGCAGACTGTCCAGA	CAGGCTCCAGAAGAAGTTGG
TBRII	GGCCAAATATCCCAAACAGA	TGGTGATGCATTTTGATGCC
RPL32 gene was used for normalization.	TCAGGTGATCTTCCCACCTC	ACCACATCCCATATCCCTCA

siRNAS-Smad7 sequences from QIAGEN

siRNA	Catalog no.	Target sequence
siRNASmad7-1	SI02636277	CTGGATATCTTCTATGATCTA
siRNASmad7-2	SI03089611	CTCCATCAAGGCTTTCGACTA

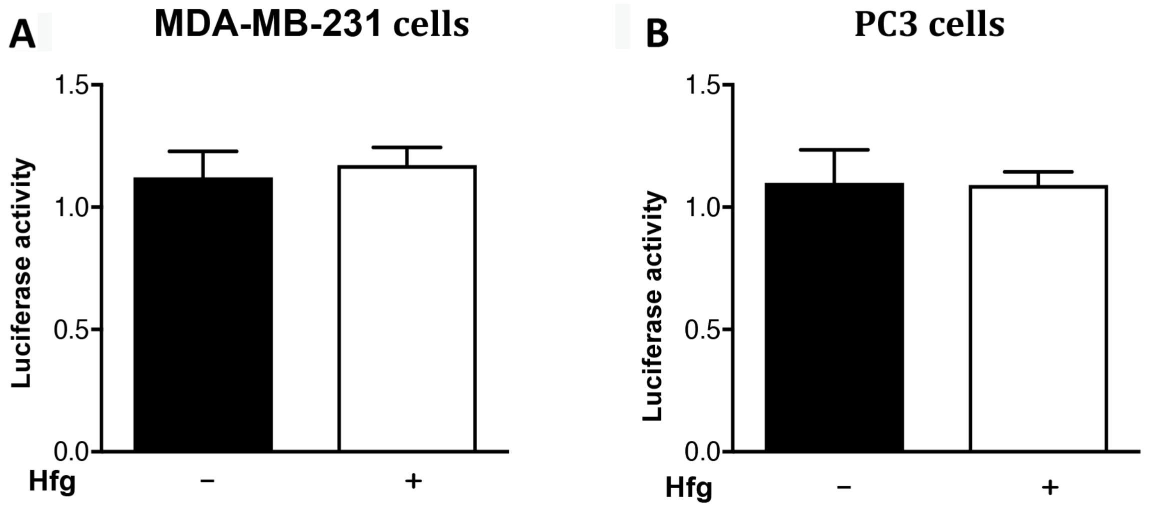

Supplementary Figure 1: CMV constitutive activated promoter is not affected by Halofuginone treatment. **(A)** MDA-MB-231 cells **(B)** and PC3 cells (were co-tranfected with a constitutively activated CMV-promoter, and phRL-CMV Renilla luciferase vector. Cells were treated with Hfg (200nM) alone for 24hrs before measuring luciferase activity.
